# Immunomodulation as a Protective Strategy in Chronic Otitis Media

**DOI:** 10.3389/fcimb.2022.826192

**Published:** 2022-03-30

**Authors:** Anke Leichtle, Arwa Kurabi, David Leffers, Markus Därr, Clara Sophia Draf, Allen Frederic Ryan, Karl-Ludwig Bruchhage

**Affiliations:** ^1^ Department of Otorhinolaryngology, Head and Neck Surgery, University of Lübeck, Lübeck, Germany; ^2^ Department of Otolaryngology, University of California, San Diego, San Diego, CA, United States; ^3^ Research Section, Veterans Affairs (VA) San Diego Healthcare System, La Jolla, CA, United States

**Keywords:** otitis media, immunology, apoptosis, TNFα, NOD-like receptors, TOLL-like receptors

## Abstract

**Introduction:**

Major features of the pathogenesis in otitis media, the most common disease in childhood, include hyperplasia of the middle ear mucosa and infiltration by leukocytes, both of which typically resolve upon bacterial clearance *via* apoptosis. Activation of innate immune receptors during the inflammatory process leads to the activation of intracellular transcription factors (such as NF-κB, AP-1), which regulate both the inflammatory response and tissue growth. We investigated these leading signaling pathways in otitis media using mouse models, human samples, and human middle ear epithelial cell (HMEEC) lines for therapeutic immunomodulation.

**Methods:**

A stable otitis media model in wild-type mice and immunodeficient KO-mice, as well as human tissue samples from chronic otitis media, skin from the external auditory canal and middle ear mucosa removed from patients undergoing ear surgery, were studied. Gene and protein expression of innate immune signaling molecules were evaluated using microarray, qPCR and IHC. *In situ* apoptosis detection determined the apoptotic rate. The influence of bacterial infection on immunomodulating molecules (TNFα, MDP, Tri-DAP, SB203580, Cycloheximide) in HMEEC was evaluated. HMEEC cells were examined after bacterial stimulation/inhibition for gene expression and cellular growth.

**Results:**

Persistent mucosal hyperplasia of the middle ear mucosa in chronic otitis media resulted from gene and protein expression of inflammatory and apoptotic genes, including NODs, TNFα, Casp3 and cleaved Casp3. In clinical chronic middle ear samples, these molecules were modulated after a specific stimulation. They also induced a hyposensitive response after bacterial/NOD-/TLR-pathway double stimulation of HMEEC cells *in vitro*. Hence, they might be suitable targets for immunological therapeutic approaches.

**Conclusion:**

Uncontrolled middle ear mucosal hyperplasia is triggered by TLRs/NLRs immunoreceptor activation of downstream inflammatory and apoptotic molecules.

## Introduction

Otitis media (OM) is one of the most common ear diseases and a serious healthcare problem. Furthermore, chronic OM is one of the most important reasons for acquired and preventable hearing loss (World Health Organization, 2004). Yet, the pathogenesis of OM is not fully understood. It is currently assumed that the etiology is multifactorial, with the innate immune system and its reaction during bacterial infections playing an essential role. *Streptococcus pneumoniae* and *Haemophilus influenzae* are the predominant bacterial pathogens of which, in the case of chronic OM, especially non-typable *H. influenzae* (NTHi) is found to be a dominant pathogen (Ngo et al., 2016). Prior viral upper respiratory infection (URI) increases OM incidence, with the middle ear (ME) exhibiting more chronic infection (Chonmaitree et al., 2015). The immune response to pathogens leads to hyperplasia, leukocytic infiltration and apoptosis of the ME mucosa (Leichtle et al., 2011; Wigand et al., 2018). Current therapy for chronic OM consists of topical and systemic antibiotics, improvement of middle ear ventilation with pressure equalization tubes (Leichtle et al., 2017) and/or surgery (Tisch et al., 2020).

The innate immune system is responsible for the rapid recognition of, and defense against, pathogens. Pattern recognition receptors (PRRs) of the ME mucosa recognize bacterial molecules, termed pathogen-associated molecular patterns (PAMPs), leading to the expression of inflammatory cytokines, chemokines, interferons and antimicrobial peptides (Takeuchi and Akira, 2010). Important representatives of the PRRs are the Toll-like receptors (TLRs) and the nucleotide-binding oligomerization domain–like (NOD) receptors (NLRs).

TLRs are membrane-bound proteins that consist of a leucine-rich extracellular domain, a transmembrane fragments and a cytoplasmic domain. They surveil both the cell surface and vesicular cell compartments for the presence of PAMPs. Recognition of PAMPs leads *via* the downstream adaptors myeloid differentiation factor 88 (MyD88) and/or TRIF (TLR domain containing adapter inducing interferon-β) to activation of NF-κB (nuclear factor kappa B) and MAPKs (mitogen-activated protein kinases), which in turn mediate the expression of important proinflammatory cytokines including interleukin-1 beta (IL1β) and tumor necrosis factor alpha (TNFα) (Kawai and Akira, 2009; Kurabi et al., 2016). The importance of the TLRs to OM resolution have been demonstrated in TLR knockout (KO) mice models, in which ME infection always results in chronic disease (Leichtle et al., 2010).

NLRs are cytoplasmic proteins that recognize intracellular PAMPs. They consist of a caspase recruitment domain (CARD), a nucleotide binding and oligomerization domain (NOD) and leucine-rich repeats. The first identified NLRs, NOD1 and NOD2, recognize peptidoglycan derivatives, that contain a diaminophilic acid (TriDAP) and muramyl dipeptide (MDP) (Inohara et al., 2005; Jeon et al., 2012). Recognition of PAMPs *via* NLRs leads *via* the adaptor RIP2 (receptor-interacting-serine/threonine-protein kinase 2) to the activation of NF-κB and MAPK pathways resulting in production of cytokines and chemokines similarly to the TLRs (Kurabi et al., 2016). NLRP3 (NLR family pyrin domain containing 3) acts differently in that it participates in the inflammasome, which processes pro-IL1β and pro-IL18 into their active forms. The importance of NLRs to ME pathogenesis has also been demonstrated using KO mouse model. which show a delayed inflammatory response to infection as well as prolonged OM with mucosal hyperplasia (Lee et al., 2019).

**Figure 1:**
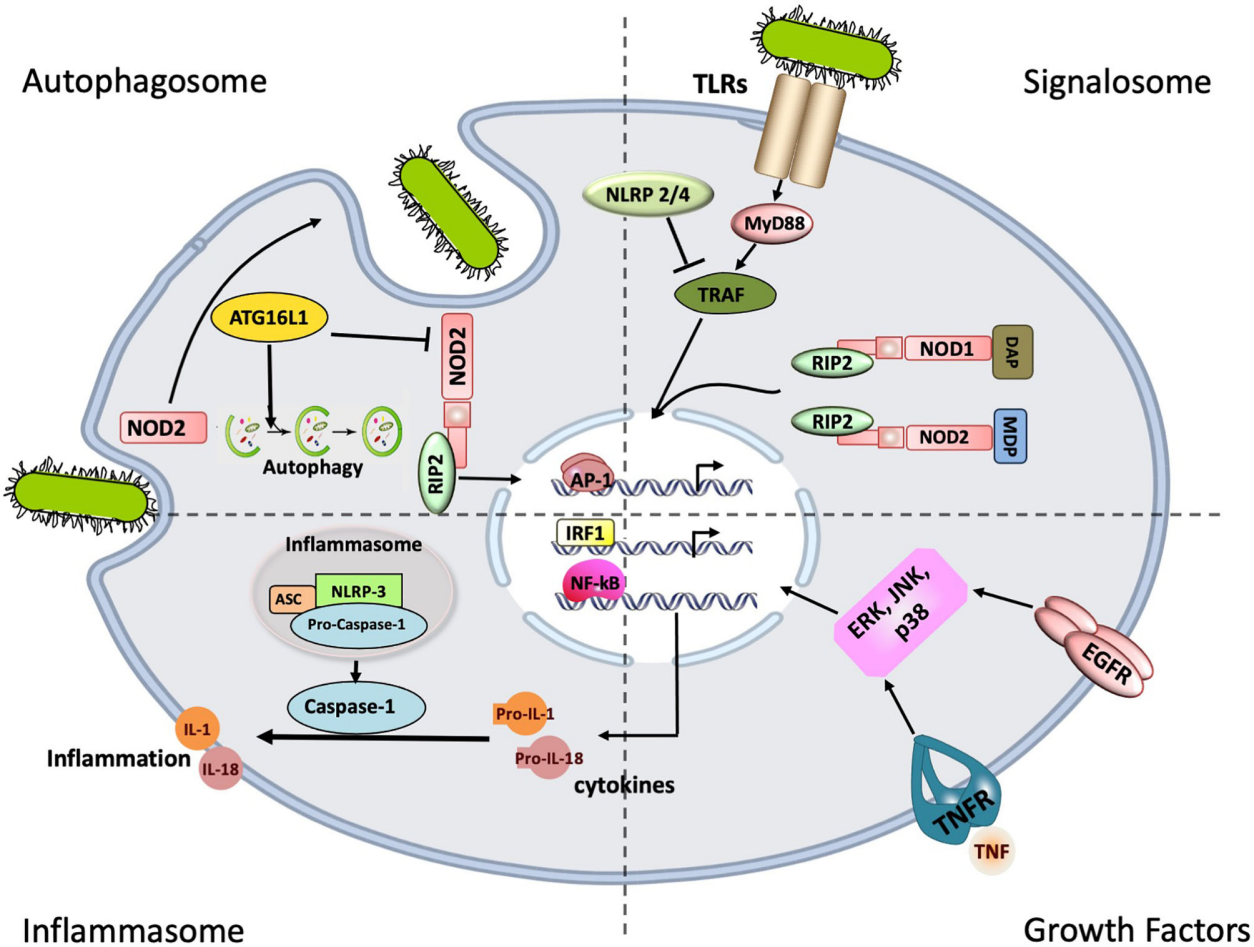
Innate immune signaling pathways implicated in bacterial Otitis Media (OM). The most common receptors involved in pattern recognition are the Toll-like receptors (TLRs), and NOD-like receptors (NRLs) of which signaling leads to the activation of Nuclear factor of kappa-B (NF-κB), the main transcription factor involved in cytokine production. Stimulation of NLRP3 leads the inflammasome to recruit and activate caspase-1, which in turn cleaves the pro-forms of IL1ß, Il18. These pro-inflammatory cytokines are in turn released in their mature/bioactive forms. The intracellular Nucleotide oligomerization domain -1 and -2 (NODs 1 and 2) can also induce inflammation through the RICK/RIP2 pathway. However, NOD2 can also induce autophagy through ATG16L1 shifting the balance of NOD2 downstream signaling. Crosstalk between the different immune elements: TLRs/NRLs, the Inflammasome and Growth factors are believed to modulate the immune responses.

These two independent innate immune systems also interact with each other. Components of the bacterial cell wall such as the muramyl dipeptides (MDP) activate both TLR2 and NOD2, while NOD2 activation *via* RICK (receptor-interacting serine/threonine protein kinase) leads to a down-regulation of the TLR2 signal cascade (Strober et al., 2006). A summary of the different innate immune pathways implicated in OM is illustrated in **Figure 1**.

NF-κB is a central regulator of many cytokine and immunoglobulin genes, and is involved in immune, inflammatory, viral, and acute phase reactions. It consists of the p50 and p65 subunits and preexists in the cytoplasm in an inactive form, complexed to its inhibitor, IκB (Kieran et al., 1990; Baltimore and Beg, 1995). PAMP-induced stimulation and degrading of the inhibitor causes NF-κB to be released and activate the transcription of proteins including ICAM1, TNFα, IL1β, IL6 and IL8 (Fujisawa et al., 1996; Johnson et al., 1997; Nassif et al., 1997; Fakler et al., 2000; Melotti et al., 2001). Several studies have noted a connection between the MyD88/NF-κB pathway and OM (Johnson et al., 1997; Nassif et al., 1997).

TNFα itself can lead to the expression of other proinflammatory cytokines, chemokines and antibacterial peptides (Vitale et al., 2007). TNFα also plays an important role in apoptosis, a critical feature of tissue remodeling during hyperplasia, recovery of normal mucosal structure during OM resolution and clearance of leukocytes from the ME. The binding of TNFα to death receptors of the TNFα receptor family induces the formation of the death-induced signaling complex (DISC) and the activation of caspase-8 and caspase-3, ultimately initiating apoptosis. This extrinsic apoptosis pathway is supplemented by an intrinsic apoptosis pathway, with mitochondria as the central component, that can be induced *via* the extrinsic pathway (Morris et al., 2018). In order to avoid excessive apoptosis, there is also an independent parallel negative feedback loop mechanism in which the activation of NF-κB by TNFα has an anti-apoptotic effect (van Antwerp et al., 1996).

In summary, innate immune responses play an important role in the timely resolution of OM. However, imbalances in the innate immune system network may lead to failure of bacterial clearance, persistent inflammation and chronic OM. A high probability of chronic OM occurs in the absence of central components of the aforementioned pathways, such as TLR, NLR, MyD88 and TNFα (Leichtle et al., 2011). Hence, these pathways are of central importance in the normal recovery from OM and in the pathogenesis of chronic OM. This study assesses the networks of the innate immune system in chronic OM, with a focus on the possibility of new immunological therapy approaches.

## Methods

### Mouse Models

TNF^-/-^, NOD1^-/-^, NOD2^-/-^, RIPK2^-/-^ mice and their age-matched C57BL/6 wild-type (WT) controls were purchased from Jackson Laboratory (Bar Harbor, ME). TL2^-/-^, TLR4^-/-^ and MyD88^-/-^ mice were originally kindly provided to us by Akira and colleagues (Hoshino et al., 1999; Miyao et al., 2006; Leichtle et al., 2009). The animals were kept under specific pathogen-free conditions (BSL2). All experiments were performed according to the National Institutes of Health Guidelines and approved by the Institutional Animal Care and Use Committee of the Veterans Affairs Medical Center in San Diego, CA.

### Bacteria

*Haemophilus influenzae* strain 3655 (nontypeable NTHi, biotype II) was used. In brief, A culture was streaked onto a chocolate agar plate and placed in a 37°C incubator overnight. two colonies were inoculated into 25 mL of brain heart infusion (BHI) media supplemented with 1 mL of Fildes enrichment (BD Diagnostic Systems) and grown for 16 hours at 37°C with shaking. The next day, the bacterial culture was spun down at 7,000 rpm for 10 minutes at 4°C and the pellet resuspended in fresh BHI media. Stock was diluted to 10^5^-10^6^ CFU/mL and 5 μL was injected per ear to induce infection in the ME of the mice (Hernandez et al., 2008).

### Animal Experiments

A total of 60 mice were examined. Groups of 6 mice per strain (TNF^-/-,^ NOD1^-/-^, NOD2^-/-^, RIPK2^-/-^, TLR2^-/-^, TLR4^-/-^, MyD88^-/-^ and WT mice) were operated under general anesthesia using ketamine rodent cocktail (Hernandez et al., 2008). A small opening was created in the surgically exposed ME bulla and both sides were inoculated on with NTHi strain described above, afterwards the intratympanic access was closed with connective tissue and the cervical access closed surgically. Untreated mice group served as a control group at time zero. Data from all mice except RIPK2^-/-^ have been published previously (Leichtle et al., 2009; Hernandez et al., 2015; Lee et al., 2019).

### Histology

ME were collected from control and NTHi treated animals on day 1, 2, 3, 5, 7 and 10, as for the KO mice on day 3 and 10. For this purpose, intracardiac perfusion with PBS was carried out under general anesthesia, followed by 4% paraformaldehyde (PFA) at the respective times. The ME of the mice were microsurgically resected, postfixed overnight with 4% PFA overnight, and decalcified in an 8% EDTA and 4% PFA solution over a 14-day period. The bullae were then processed in 30% sucrose followed by OCT media and cut as cryosections. For tissue histology, the ME bullae were embedded in paraffin, sectioned at 7 μm and stained with hematoxylin-eosin (H&E). Sections from the same region of each middle ear were digitally recorded, and the mucosal thickness and the percentage area of the middle ear lumen occupied by inflammatory cells were determined at standard locations. The data were not normally distributed and were analyzed using the Kruskal-Wallace nonparametric ANOVA. Differences between groups were considered significant at p <0.05.

### Gene Microarray

Affymetrix gene arrays were used to quantify relative ME gene expression profiles during the course of OM. The MEs of 40 mice per time point were inoculated bilaterally with NTHi. The ME mucosa and exudate were harvested from 20 mice and pooled at each of the following time points: 0 hours (0h, no treatment), 3h, 6h, 24h, 2 day (2d), 3d, 5d and 7d. Total RNA was extracted by homogenizing the tissue in TRIzol (Life Technologies, Carlsbad, CA) and reverse transcribed to generated biotinylated cRNA library that were hybridized onto the MU430 2.0 microarrays per time point sample according to the manufacturer’s protocol. This procedure was duplicated for each time point using the other 20 mice. Hence, each data set represents 2 independent biological replicates and 4 Affymetrix arrays. The raw data were median normalized using Variance-modeled posterior Interence Approch (VAMPIRE) and the specific gene transcript fold-level changes were assessed using Genespring GX 7.3 (Agilent Technologies, Santa Clara, CA). Detailed methods are provided (Hernandez et al., 2015).

### Cell Culture

The human middle ear epithelial cell line (HMEEC) (Chun et al., 2002) was a generous gift from David J. Lim (Department of Head and Neck Surgery, University of California Los Angeles, CA, USA). According to the protocol (Woo et al., 2014), the cells were maintained in a 1∶1 mixture of DMEM and bronchial epithelial basal medium (Lonza, Walkersville, MD) supplemented with bovine pituitary extract (50 µg/ml), hydrocortisone (0.5 µg/ml), hEGF (0.5 ng/ml), epinephrine (0.5 µg/ml), transferrin (10 µg/ml), insulin (5 µg/ml), triiodothyronine (6.5 ng/ml), retinoic acid (0.1 ng/ml), gentamicin (50 µg/ml), and amphotericin-B (50 ng/ml). The cells were cultured at 37°C under 5% CO2 and 95% air atmosphere. For immunomodulation, the HMECCs were stimulated with NTHi, TNFα, MDP, Tri-DAP, SB203580 (p38 MAP Kinase Inhibitor) and CHX (Cycloheximide) alone or in combination for 6, 24, 48 and 72 hours and qPCR or MTT Assay was performed. Cycloheximide was used to inhibit protein synthesis in eukaryotic cells and initiates apoptosis as a positive control, since it works rapidly and strongly *in vitro*.

### Human Tissue Samples

After giving consent, samples of chronic OM (COM) (n=20), the external auditory canal skin (EAS) (n=20) and healthy middle ear mucosa (n=20) were taken from patients during middle ear operations at the ENT department of the University of Lübeck (Germany) (see **Table S1** for further clinical details). The median age of the patients with COM was 51.8 years (range, 22 to 82 years). All 20 patients had surgery due to COM with hearing loss. The tissue samples for healthy controls (n= 20 middle ear and n= 20 EAS) were collected from 21 patients with a median age of 56.4 years (range, 30 to 80 years), undergoing surgery for hearing rehabilitation with a cochlear implant, a vibrant soundbridge implant, tympanoscopy or a second-look surgery. All samples, if not processed immediately, were kept in liquid nitrogen and stored at -80° C according to the protocol of our previous publications (Leichtle et al., 2015; Leichtle et al., 2021). All protocols were approved by the ethics committee of the University of Lübeck (10-039, 20-434). All clinical examinations were carried out according to the principles of the Declaration of Helsinki (1964).

### Quantitative Real-Time PCR

The procedure was carried out according to the protocol of our previous publications (Lai et al., 2009; Leichtle et al., 2010). In brief, the mRNA was extracted from the human tissue samples (COM: n = 20, ear canal biopsies: n = 20 and healthy middle ear mucosa: n = 20) or from the HMECC using RNeasy Mini Kits (Qiagen, Mississauga, ON, Canada). The amount of RNA was measured with a spectrophotometer. According to the manufacturer’s protocol, 0.5 μg of total RNA was converted into cDNA using the first strand cDNA synthesis kit (Fermentas, St.Leon-Rot, Germany). After the reverse transcription reaction (RT), all samples were diluted 1: 4 in ddH2O and subjected to real-time PCR analysis with Maxima SYBR Green QPCR Master Mix (Fermentas, St. Leon-Rot, Germany). 0.3 µM gene-specific primers (TNF, IL1-β, IL8, NOD1, NOD2, RIPK2, TLR2, TLR4, BID, CAS7, CASP3) and GAPDH, for a total reaction volume of 25 µl. For all targets, the cycling conditions were: 50°C for 2 minutes, 95°C for 10 minutes, followed by 40 cycles each consisting of 95°C for 15 seconds, 60°C for 30 seconds, and 72°C for 30 seconds. Integration of the SYBR Green dye into the PCR products was monitored using the ABI PRISM 7000 sequence detection system (Applied Biosystems, Carlsbad, CA, USA). The quantification of gene expression was determined by the comparative ΔΔC_T_ method. The target gene expression in the test samples was normalized to the endogenous reference GAPDH level and was reported as the fold difference relative to GAPDH gene expression (Leichtle et al., 2015; Leichtle et al., 2021). All measurements were performed in triplicate and three independent experiments performed for each gene target.

### Immunohistochemistry

For LSAB (Labeled (Streptavidin-Biotin) staining, paraffin-embedded, formalin-fixed tissue sections were deparaffinized and re-hydrated in xylene, ethanol and TBS. Endogenous peroxidases (15 min incubation in 3% H_2_O_2_) and endogenous biotin (Avidin/Biotin Blocking Kit, Vector Laboratories, Burlingame, CA) were blocked. For antigen retrieval, sections were incubated in Proteinase K (DAKO, Carpinteria, CA) for 7 min, blocked with 1% BSA in PBS and incubated with the primary rabbit anti-Nod1 (1:200, Imgenex IMG 5739), anti-Nod2 (1:600, Santa Cruz, sc 56168), anti-RIPK2 (1:200, Abcam, ab 75257), anti-caspase3 antibody (1:100, cell signaling) and anti-cleaved (c) -Caspase3 antibody (1:100, Cell Signaling) in PBS overnight. Washed in 0.1% BSA, washed in PBS and treated with biotin sheep anti-rabbit secondary antibodies (DAKO) and the AEC peroxidase substrate kit (Vector Laboratories) according to the manufacturer’s instructions. Positive controls were performed according to the protocol. Negative controls without antibody were used for every experimental epitope target.

### *In Situ* Apoptosis

The apoptosis rate in OM samples with cellular effusion was determined by Image-iTTM LIVE Red Caspase-3 and -7 Detection Kit (Thermo Fisher Scientific Inc., Schwerte) according to the manufacturer’s protocol. The removed tissue was directly covered with the 30X FLICA reagent working solution in a dilution of 1:30, incubated for 60 min protected from light at room temperature and rinsed with the 1x wash buffer 1:10. Counter-staining of the cell nucleus was performed with 1mM of Hoechst 33342 (1:1000) and 5mM SYTOX green for 10 min below room temperature. The sample was washed twice with 2 ml of 1X wash buffer. The sample cover-slipped and 2 ml 1X wash buffer and evaluated under the confocal fluorescence microscope (LSM 510, Carl Zeiss and DM IRB, Leica).

### MTT Assay

Cellular metabolic activity was determined by a quantitative colorimetric 3-(4,5-dimethylthiazol-2-yl)-2,5-diphenyl tetrazolium bromide (MTT) assay. This assay determines viable cell numbers based on the mitochondrial conversion of MTT. Approximately 5 to 10 × 10^3^ cells were dispersed into each well of a 96-well plate and cultivated with different stimuli (NTHi, TNF, Tri-DAP and MDP). 10 µL of MTT dye (5 mg/mL; Sigma-Aldrich) was added for 1 hour at 37°C and then100 µL of MTT stop solution was added to stop the reaction (Sigma-Aldrich). After gently shaking for 24 hours, absorbance was measured at 570 nm using a Benchmark Plus microplate reader (BioRad, Hercules, CA, USA). All measurements were performed in triplicate and three independent experiments were performed for the assay.

### Statistics

Data are reported as arithmetic means ± standard deviation. ANOVA with Bonferroni correction for multiple tests was performed with StatView and GraphPad Prism for normally distributed data. The percentages of the ME area covered by leukocytes were compared by the non-parametric Mann-Whitney U-test. Differences between groups were considered significant at p <0.05. The two ears from each mouse were treated as independent samples since they were found to be independent form each other (Ebmeyer et al., 2005). Sex contingency was compared with a two-sided Fisher’s Exact Test.

## Results

### Gene Microarray

**Figure 2:**
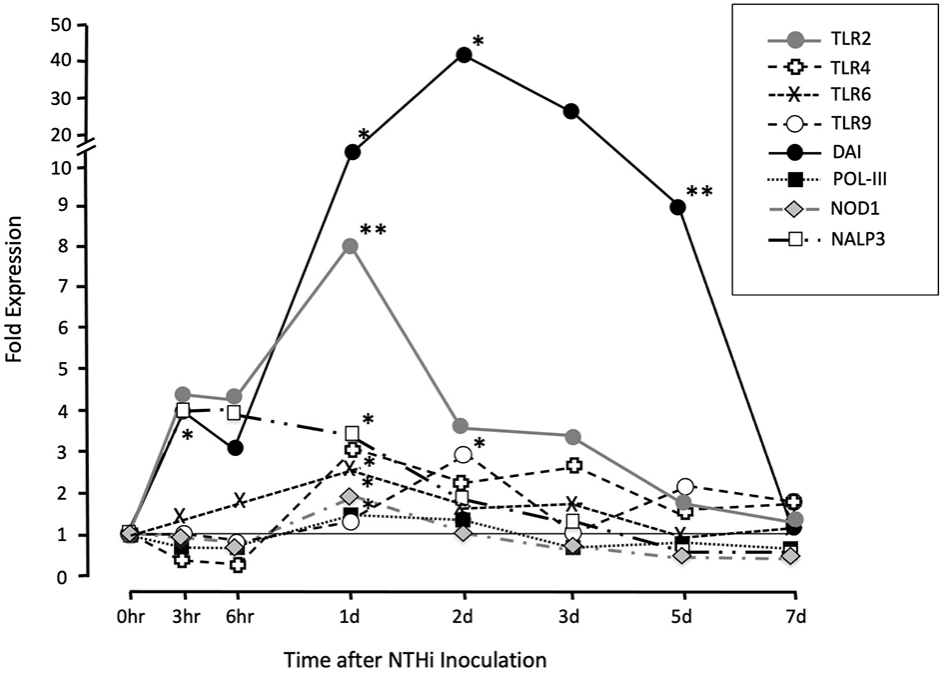
Assessment of genes involved in patter recognition during a complete episode of acute NTHi-induced OM in the mouse, from initiation to recovery, evaluated by gene arrays. The cell surface TLR2, TLR4 and TLR6 were expressed in sentinel mucosa and highly upregulated in early time points (3hrs to 24hrs). The intracellular PRRs TLR9 was upregulated at later time points (2d). Meanwhile, NLRP3 expression peaked between (3hrs to 24hrs). The DNA-dependent activator of IFN-regulatory factors (DAI) and DNA sensor RNA polymerase (Pol-III) expression peaked later at day 2 onwards. *p < .05; **P < .01

Gene arrays were utilized to provide quantitative evaluation of key innate immune PRR expression levels within the ME. We followed gene expression throughout the course of an episode of OM after NTHi inoculation in the mouse. A variety of receptors that respond to molecules produced by pathogens were evaluated (**Figure 2**). These include the TLR (TLR2, TLR4, TLR6, TLR9) and the NLR (NOD1 and NLRP3) families, as well as several receptors that recognize foreign DNA. Part of these data have been published previously in separate reports (Hernandez et al., 2015; Lee et al., 2019), and are aggregated here with our gene analysis. Most dramatically TLR2, NLRP3 and the IFN regulatory factor (DAI), which functions as a DNA sensor, were rapidly upregulated within 3-6 h post bacterial inoculation. DAI expression remained day 7, when the infection resolved. Detailed data on fold change ranges and statistics are presented in the **Supplementary Table S2**.

### Mucosal Hyperplasia

**Figure 3:**
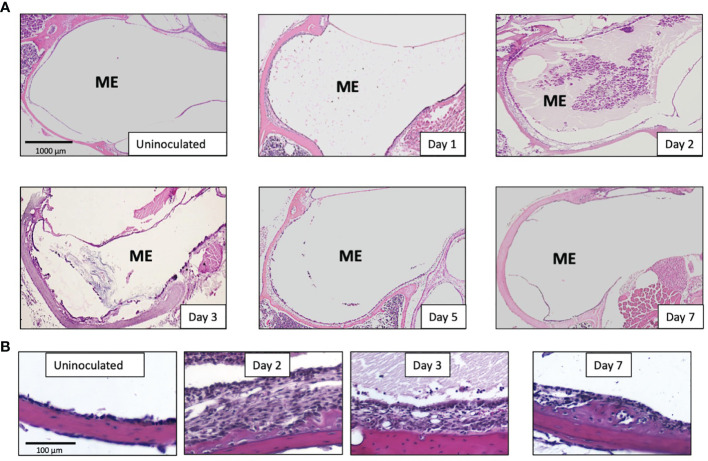
Pathophysiology of the middle ear during a NTHi induced OM in WT mice (C57/BL6). **(A)** OM time course showing cross-sections of the ME bulla and revealing that the ME mucosal hyperplasia and cellular infiltration peaks at 2-3 days after bacterial inoculation and recover by 5-7 days. Cellular infiltrate are evident on day 1, peak by day 2 and return to baseline by day 7 **(B)** Middle ear mucosa thickening is seen at a higher magnification (10x) at the indicated OM time points.

**Figure 4:**
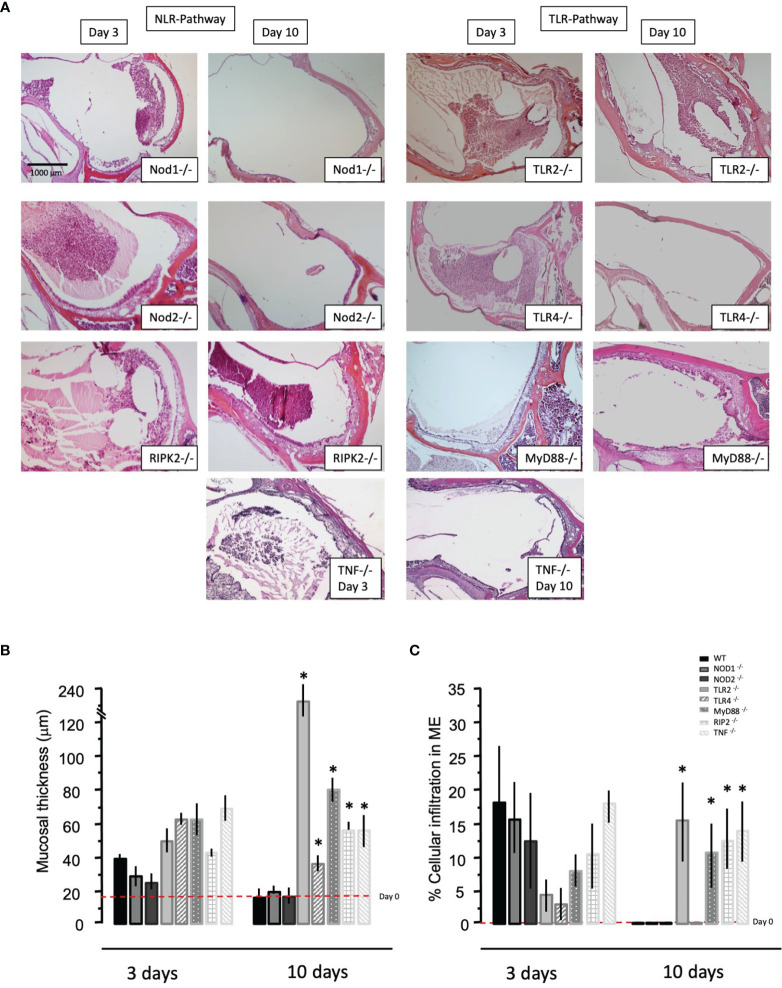
Representative histological changes at 3 and 10 days post NTHi challenge seen in middle ears of different immunodeficient KO mice. **(A)** Considerable inflammation in the ME space (filling the cavity with fluid and inflammatory cells) is observed on day 3 and by day 10 persistent mucosal hyperplasia and cell recruitment is noted in some of the immunodeficient KO animals. **(B)** Quantitative analysis of mucosal thickness and **(C)** the % area of the middle ear cavity covered with inflammatory cells in KO mice. (n=6 ears, *p < 0.05).

Having observed that NTHi infection regulates the different innate immune receptors and key adaptor components at the gene level using microarrays, we investigated the ME response to NTHi infection in mice lacking TLRs (TLR2, TLR4), NRLs (NOD1, NOD2), the obligatory immune adaptors (MyD88, RIP2), and the major innate immune effector gene TNFα, and compared the ME histopathology to wild-type C57 controls. **Figure 3** shows the histology of OM in WT type mice after NTHi inoculation. The ME mucosal hyperplasia is noted within 24h after NTHi exposure and reaches a maximum at 2-3 days, with leukocytes infiltration peaking about the same time. After which, the hyperplastic response transitions to recovery and healing, with a return to normal thickness observed by day 7. Infection of WT mice by NTHi produces an acute OM lasting only a several days. However, mice with innate immune gene deficiencies exhibit more persistent OM, and for some knockouts lasted several weeks (**Figure 4**). The data, published previously in separate reports (Hernandez et al., 2008; Leichtle et al., 2009; Leichtle et al., 2010; Lee et al., 2019), are aggregated here and compare with mouse models of RIPK2 as well. **Figure 4A** reveals the OM pathogenesis at 3 days and 10 days post NTHi inoculation revealing that ME mucosal healing was altered in the absence of these innate immune molecules. **Figure 4B** illustrates quantitative evaluations of the mucosal thickness in the KO mice plus levels of leukocyte infiltration of the ME cavity. We observed that the mucosal thickness and leukocyte clearance were delayed in the immune deficient mice compared to that seen in WTs, especially in TLR2-deficiency by day 10. Meanwhile, the NLR pathway, in the absence of NOD2 and NOD1, responded hyposensitively at day 3. Compared to the WT phenotype, mucosal growth was lower in the NOD1 and NOD2 KO mice. The percentage of ME lumen occupied by infiltrating leukocytes was also lower on day 3 in theses mice.

### Immunoregulation in Human OM. Expression of Innate Immune Receptors in Human COM

**Figure 5:**
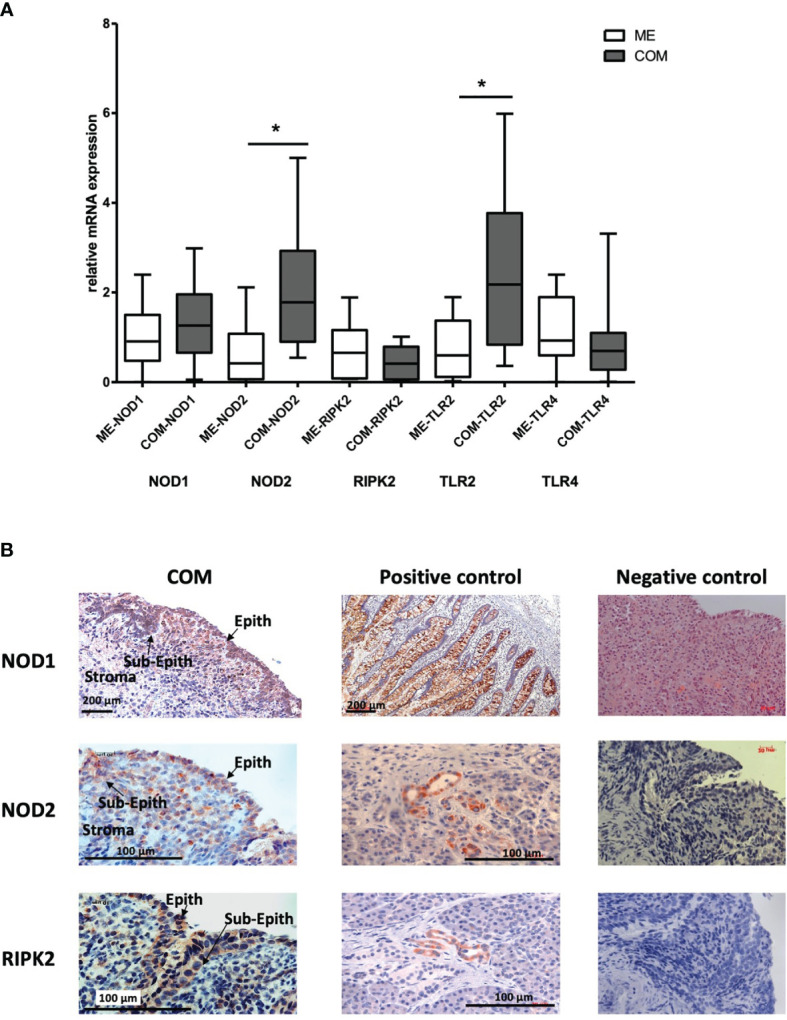
**(A)** Gene expression of Innate Immune Receptors in human COM. mRNA expression of NOD1, NOD2, RIPK2, TLR2 and TLR4 in healthy middle ear samples (ME) and COM relative to GAPDH. Significantly increased gene expression of NOD2 and TLR2 in COM compared to healthy middle ear tissue. For normalization, the housekeeping gene GAPDH. N = 20 each; GraphPad Prism with an unpaired t-test, *p < .05. **(B)** Protein expression of NLR Immune Receptors in human COM. NOD2, NOD1 and RIPK2 could be localized immunohistochemically mainly intra- and subepithelial in COM. The right column displays their positive control in small intestine (NOD1) and in pancreas (NOD2 and RIPK2). Orange/brown represents the target molecule NOD1, NOD2 and RIPK2, blue represents nuclei. Scale is shown by scale bar insert.

Using RT-PCR, the gene expression of the innate immune receptors NOD1, NOD2, RIPK2, TLR2 and TLR4 in human samples of COM were investigated (**Figure 5A**). and compared to healthy middle ear tissue. For normalization, the housekeeping gene GAPDH was used. Significantly increased gene expression of NOD2 and TLR2 was observed in COM. N = 20 each; GraphPad Prism with an unpaired t-test, p<0.05. The mRNA expression of NOD2 and TLR2 was significantly increased compared to healthy tissue samples of the ME. However, the gene expression of the patients showed large variability. The gene expression of NOD1 was increased but not significantly expressed when compared to healthy ME tissue. Meanwhile, RIPK2 and TLR4 were constitutively expressed, without up- or down-regulation. Overall, the results show an inhomogeneous gene distribution of the individual patients with high variability, which was related to the different clinical expression of COM and surgical findings intraoperatively (see **Table 1** and **Supplementary Table S1**). For protein expression, immunohistochemical staining using immunoperoxidase (DAB) on cryosections was performed detecting the three NLR-pathway receptors in human COM, which is a novel finding. **Figure 5B** shows the expression of NOD1, NOD2 and RIPK2 proteins in human OM and their positive controls. All three receptors could be readily detected intra- and subepithelial in COM. Similar to the gene expression results, NOD1 and NOD2 protein expression in COM was visibly elevated compared to RIPK2 protein expression. Immunohistochemistry also revealed that NOD1 is localized primarily in the epithelial layers of the middle ear tissue, whereas NOD2 and RIPK2 seems to be present in the basal epithelial layer and subepithelium.

**Table 1:** Clinical and demographic information from patients utilized in this study.

	COM	Healthy	*p value*
	n =20	n=21	significant at *p* < .05
**Mean age in years (range)**	51.8 (22-82)	56.4 (30-80)	0.19
**Female Gender**	12	8	0.22
n (%)	60%	38%	
**Hearing loss**	20	15	0.51
n (%)	(100%)	(71%)	
**Vertigo**	6	2	0.48
n (%)	(30%)	(10%)	
**Diagnoses/Procedure**			
COM	16	–	
COM with facial palsy	2	–	
COM with mastoiditis	2	–	
Acute suditas for tympanoscopy	–	4	
Cochlear implantation	–	8	
Vibrant Soundbridge implantation	–	4	
Second-Look	–	4	
Meatoplasty	–	1	

### Gene Expression of Downstream Genes TNF and IL8 in Human COM

**Figure 6:**
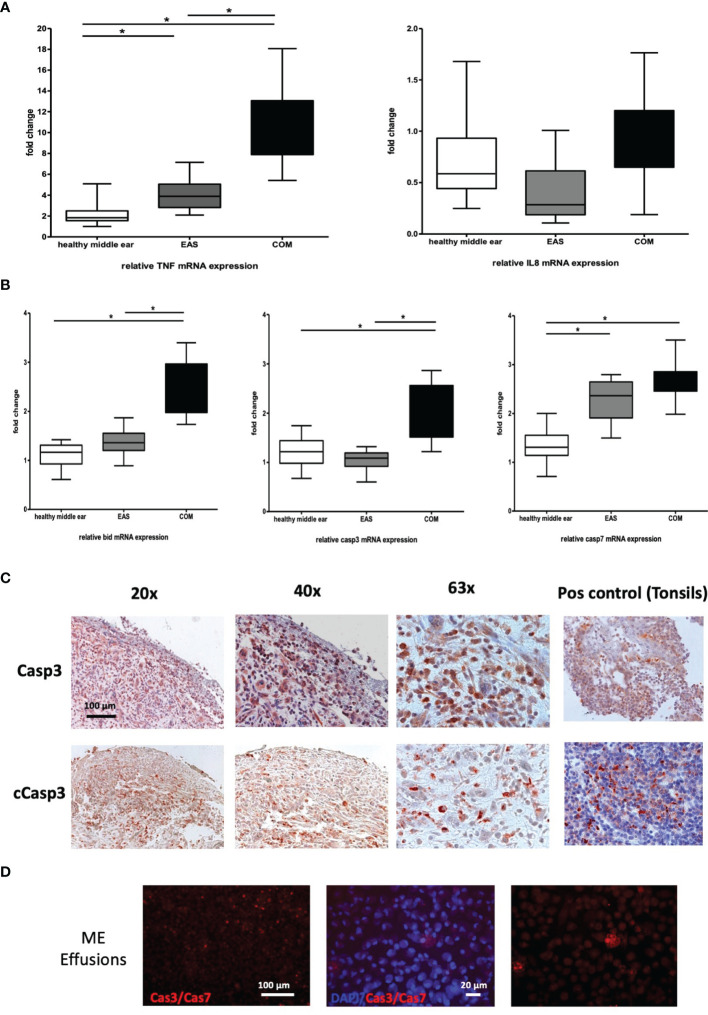
**(A)** Gene expression of TNF and IL8 in human COM. TNFα (left) and IL8 (right) mRNA expression in healthy middle ear samples, external auditory skin (EAS) and COM relative to GAPDH. Significantly increased gene expression of TNFα in COM compared to EAS and healthy middle ear samples. No significant mRNA regulation of IL8. **(B)** Gene expression of apoptotic genes in human COM. Bid, Casp3, and Casp7 mRNA expression in healthy middle ear samples, external auditory skin (EAS) and COM relative to GAPDH. Bid and Casp3 mRNA expression in COM is significantly increased to EAS and healthy middle ear samples (left and middle). Significant upregulation of Casp7 in EAS and COM to healthy samples (right). Normalization with GAPDH; N = 20 each; GraphPad Prism with an unpaired t-test, *p < .05. **(C)** Expression of apoptotic proteins. Immunohistochemistry by immunoperoxidase of Casp3 and cCasp3 as brownish-orange staining in human COM. Casp3 is broadly stained in the epithelium and subepithelial, whereas active cCasp3 protein expression shows staining mainly in the subepithelial layers. 20x, 40x and 63x magnification. **(D)** Caspase3 and 7 livestain in COM with cell effusion shows a disseminated red staining of single cells mainly subepithelial. 20x and 63x magnification.

For a more detailed gene evaluation between the different human tissue, we investigated the relative expression of the effector genes TNF and IL8 in healthy ME mucosa; n=20, external auditory skin (EAS); n=20, and COM; n=20, relative to GAPDH (**Figure 6A**). Expression of TNFα mRNA in COM was significant increased compared to the external auditory skin and healthy ME mucosa. The individual gene expression levels in COM show a significantly broader distribution compared to the other tissue samples. The mRNA gene expression was most homogeneous in healthy ME mucosa. In contrast, the IL8 gene expression in the COM show no significance change from external auditory skin or healthy ME mucosa, suggesting that IL8 production was similar in all three samples.

### Gene Expression of Apoptotic Genes in Human COM

Given the upregulation of TNF gene expression in COM, the gene profiles of extrinsic and intrinsic apoptotic genes were examined. Relative mRNA expression of Bid, Casp3 and Casp7 were respectively analyzed relative to GAPDH in same as above. The gene expression of Bid (left) and Casp3 (middle) in COM was significant increased compared to the healthy ME mucosa and EAS, displaying a broad distribution (left). A significant upregulation of Casp7 in EAS and COM compared to healthy samples (right) could be detected as well (**Figure 6B**).

### Expression of Apoptotic Proteins in Human COM

The localization of the apoptotic protein Casp3 and cleaved-Casp3 (cCasp3) were analyzed using immunohistochemical staining in human COM (**Figure 6C**). Both proteins were easily detected in the epithelium and subepithelial in COM. Casp3 showed distinct staining intra- and subepithelially. Active cCasp3 protein expression was mainly located in the subepithelial layer. Negative controls, without antibody, and positive controls (using tonsil tissue), were performed to validate the epitope staining. To detect active caspases, the Image-iTLIVE Detection Kit Red for Caspase-3 and -7 was used on fresh samples from COM with cell effusion directly excised from surgery (**Figure 6D**), based on a fluorescent inhibitor of caspases (FLACA) method. The remaining red fluorescent signal showed a direct measure of the amount of active caspase present at the time the inhibitor was added. Caspase3 and 7 activity was detected in COM middle ear effusions, as disseminated red staining of single cells.

### Immunomodulation in the Human Middle Ear Epithelial Cell Line (HMEEC)

**Figure 7:**
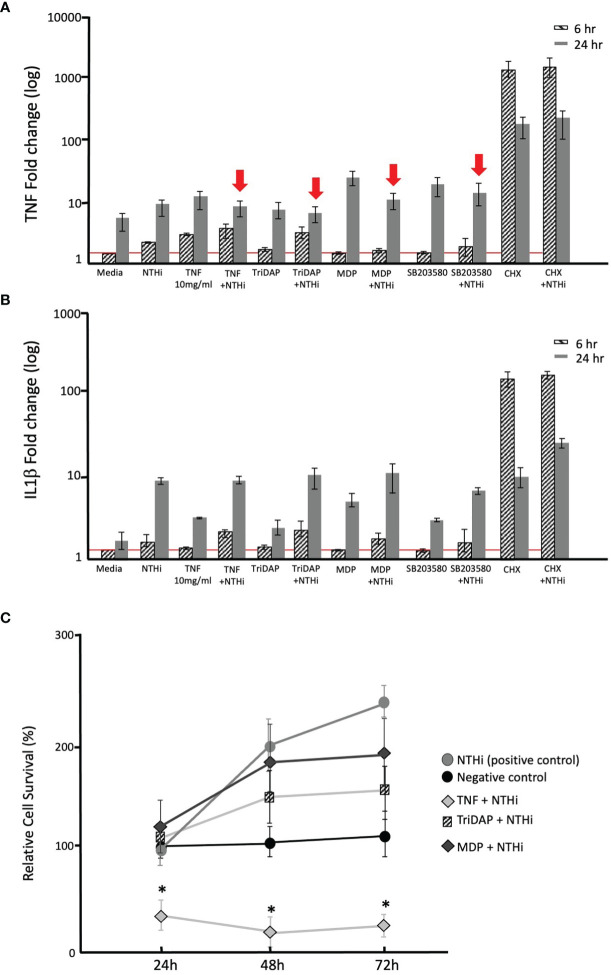
Immunmodulation in the Human Middle Ear Epithelial Cell Line (HMEEC). Gene expression of TNF **(A)** and IL1β **(B)** in HMECC at 6h and 24 h after stimulation with NTHi, TNF, Tri-DAP, MDP, SB203580, CHX or double-stimulation, was evaluated using qPCR. For normalization, the housekeeping gene GAPDH was used. GraphPad Prism with an unpaired t-test, *p<0.05. **(A)** Increased gene expression of TNFα after stimulation with NTHi, TNFα and CHX or their double stimulation with NTHi at 6h in HMEEC compared to medium. At 24h induction of all stimuli or their double stimulation with NTHi in HMEEC compared to medium. However, double-stimulation with NTHi and TNFα, or Tri-DAP, or MDP, or SB203580 compared to the direct single stimuli molecule showed hyporesponse at 24h, with a reduction in TNFα expression (red arrows). **(B)** Significantly increased gene expression of IL1β only after stimulation with single stimulation with NTHi, or CHX; or double stimulation with NTHi and TNFα, TriDAP, MDP and CHX at 6h compared to controls. At 24h significant IL1β induction was observed under all conditions compared to controls. However, double-stimulation with NTHi and TNFα, Tri-DAP, MDP or SB203580 responded with no change in IL1β gene expression when compared to stimulation with NTHi alone at 24h. **(C)** Effects of NTHi, TNF, TriDAP, MDP and their combinations on the cellular viability of cultured HMEECs assessed by MTT assay. TNFα had a significant effect on cellular viability after incubation with NTHi Significant difference (*p* < 0.05) is marked with *.

Following our observations regarding the involvement of TLR and NODs in COM in murine models (*in vivo*), and the human tissue data implicating TLRs and NODs in inflammation and apoptosis, we set out to explore the functional stimulation of these receptors using HMEEC cells *in vitro*. **Figures 7A, B** shows the gene expression of TNFα and IL1β in HMEEC at 6h and 24h post stimulation with NTHi, TNF, Tri-DAP, MDP, SB203580, CHX or double-stimulation. (**Figure 7A**) At 6h, the mRNA expression of TNFα in HMECC stimulated with NTHi, TNFα and CHX or their double stimulation with NTHi was significantly increased compared to medium alone (negative control). As well, TNFα expression at 24h showed a significant induction after treatment with NTHi, TNFα, Tri-DAP, MDP, SB203580, CHX or their double stimulation with NTHi in HMEEC compared to medium control. The strongest increase displayed was using MDP and CHX, representing the TLR-NOD2 and apoptosis pathways. Interestingly double-stimulation with NTHi and TNFα, or Tri-DAP, or MDP or SB203580 responded in contrast to their single molecule stimulation with a decrease of TNFα gene expression at 24h (depicted with red arrows), in **Figure 7B**. Compared to TNF, the mRNA expression of IL1β in HMECC at 6h was only induced after stimulation with CHX, or double stimulation with NTHi and TNFα, TriDAP, MDP and CHX compared to the media alone control. However 24 h after stimulation, all stimuli, except SB203580 (a p38 MAP Kinase Inhibitor), caused a significant increase in mRNA expression of IL1β compared to the negative control (media only). The strongest increase in single stimulus displayed were again NTHi plus MDP or CHX with apoptosis induction. In contrast to TNFα, double-stimulation with NTHi and TNFα, and NTHi + Tri-DAP or + MDP resulted in no remarkable change in IL1β expression. Moreover HMEEC single or double stimulation with SB203580 resulted in lower IL1β expression at 24h compared to the NTHi stimulus alone.

### Cell Viability in HMEEC After Immunostimulation

MTT assays were used in order to determine whether specific immunomodulation led to an altered cellular viability of HMEEC (**Figure 7C**). Samples treated with NTHi, TNFα, TriDAP and MDP, and their combinations were analyzed to quantify the cellular metabolic activity as an indicator of cell viability at 24, 48 and 72 hrs. The addition of NTHi to cells stimulated cellular proliferation with an increase in % cells. Single stimulation with TNFα, NOD ligands MDP and TriDAP did not have an effect on cell viability (negative control remained at 100%). Double Stimulation of the HMEECs with TNFα and NTHi decreased the cellular proliferation significantly. In response to the double stimulation with MDP or TriDAP with NTHi, no effect was observed on cell viability after 72h monitored.

## Discussion

Animal studies have shown that innate immunity plays an important role in the pathogenesis of OM. Pathogenic OM features including inflammation, mucosal hyperplasia and leukocyte infiltration are initiated within hours after ME infection, consistent with innate immune response to pathogens (Hernandez et al., 2008). The essentially immediate innate immune response to pathogens suggests that many innate immune genes are constitutively expressed by ME cells. This has been confirmed by single-cell RNA-Seq of the normal mouse ME (Ryan et al., 2020). Genes encoding the TLR and NLR families, as well as several receptors that recognize foreign DNA, are also significantly up-regulated in the ME during animal OM (**Figure 2**).

In addition to pathogenesis, innate immunity plays a significant role in OM resolution (Massa et al., 2021). In the normal child, OM typically recovers within a few days, too soon for adaptive immunity to be engaged (Leichtle et al., 2011). Animal studies have shown that imbalances in innate immune activity can lead to failure of bacterial clearance and hence COM (Hernandez et al., 2008; Leichtle et al., 2009; Leichtle et al., 2010; Underwood and Bakaletz, 2011; Kurabi et al., 2016; Lee et al., 2019). In humans, several studies have associated polymorphisms in several innate immune genes with proneness to COM (Toivonen et al., 2017; Emonts et al., 2007; Ilia et al., 2014; Nokso-Koivisto et al., 2014). Moreover, ME responses to bacteria are disabled in mice deficient in innate immune receptors or effectors (**Figure 4**). Infected with the otopathogen NTHi, uncontrolled mucosal growth and leukocyte infiltration, combined with significantly pronounced inflammatory components, are observed in the ME, similar to the histopathological features found clinically during ME surgeries for OM. Especially, mice deficient in the innate immune receptors TLR2 and NOD2, their adaptor molecules Myd88 and RIPK2, and downstream TNFα demonstrate highly persistent OM after NTHi infection (Hoshino et al., 1999; Leichtle et al., 2009; Leichtle et al., 2010; Lee et al., 2019). Apoptosis also contributes to OM recovery. For example, OM pathogenesis is enhanced in mice deficient in Fas-mediated apoptosis (Rivkin et al., 2005).

Recent studies in other systems have demonstrated that a functional immune system is important in keeping inflammatory responses and apoptosis under control (Man and Kanneganti, 2016; Birkle and Brown, 2021). In the infected mouse ME, negative regulators of inflammation are expressed rapidly and simultaneously with pro-inflammatory innate immune effectors (Hernandez et al., 2015). This finding underscores the importance of host control over the pathogenic effects of inflammation mediated by innate immune response to bacteria.

Given prior ME data from the mouse models, association of human innate immune gene polymorphisms with OM proneness, and the documented expression of genes in the human ME during OM (**Figures 2**, **4**), the network of TLRs, NLRs, and their upstream and downstream signaling pathways during OM take on translational significance. In ME tissue from OM patients we found that TLR2 and NOD2 mRNA expression was significantly induced during COM compared to healthy ME tissue, although variability between patients was high (**Figure 5**). TLR2 upregulation in human COM tissue has been described previously by other research groups at the gene and protein levels (Hirai et al., 2013; Jesic et al., 2014; Kaur et al., 2015; Jung et al., 2021). For example, TLR2 expression was higher in the inflamed mucosa of COM MEs compared with the normal control MEs (Jesic et al., 2014), with significantly more TLR2 mRNA in bacteria-positive compared to bacteria-negative MEs (Kaur et al., 2015). Similar differences in ME bacterial load might explain the inter-individual variability in innate immune receptor expression in our COM patient data. In contrast to COM, no difference in TLR2 mRNA or protein levels was observed in prior studies of patients suffering from chronic suppurative OM (CSOM) (Si et al., 2014; Tuoheti et al., 2021). It is possible that the latter result reflects differences in response to the distinct pathogens of this disease, which typically originate from the external auditory canal after tympanic membrane rupture. It is perhaps more likely that a lack of TLR2 response disposes toward this more serious OM type. Certainly the association of distinct innate immune gene alleles with COM susceptibility, as discussed above, argues for a genetic basis of a portion of OM phenotypic variation.

Compared to cell-surface expressed TLR receptors and its downstream signaling molecules role in OM (Jung et al., 2021), less is known regarding the importance of the cytoplasmic PRRs in sensing and responding to bacterial infections in the ME (Kurabi et al., 2016). In this study, we identified NOD2 as significantly upregulated at the gene and protein level, where it was localized intra- and subepithelially in COM (**Figure 5**). NOD2 upregulation is consistent with activation of this receptor, which would require internalization of bacterial PAMPs or bacteria themselves. Internalization of bacteria into phagocytes clearly occurs in OM. However, both *Streptococcus pneumoniae* (Novick et al., 2017) and nontypeable *Haemophilus influenzae* (Hardison et al., 2018) are known to invade epithelial cells under circumstances that occur in OM, and NOD2 activation could also occur epithelially.

We observed that TNFα was significantly upregulated in COM compared to healthy ME mucosa and EAS, compared to the absence of upregulation for IL8 (**Figure 6A**). TNF is well known as a key regulator of not only inflammation but also apoptosis (Chen and Goeddel, 2002; Aggarwal, 2003; Ebmeyer et al., 2011). Indeed, evidence of apoptosis was apparent in our OM patient samples. Expression of the extrinsic and intrinsic apoptotic genes Bid, Casp3 and Casp7 was significantly increased compared to the healthy ME mucosa. A distinct differential localization of Casp3, cCasp3, and Casp7 was observed in live-stained mucosal epithelium and subepithelial in COM (**Figures 6C, D**). In comparison, studies of cholesteatoma (COM epitympanalis) found no regulation of caspase signaling by various research groups (Miyao et al., 2006; Klenke et al., 2012; Leichtle et al., 2021). Cellular substrates of apoptosis in the ME include remodeling or recovering mucosal cells and immunocytes at the end of their life cycle.

We evaluated innate immune stimulation and cell viability using HMEEC, to assess specific immunomodulation and protection strategies (**Figure 7**). Gene expression of TNFα and IL1β, induced by TLR2- and NOD2- activating stimuli, indicated rapid TLR signaling and apoptosis activation (**Figure 7A**), followed by later IL1β and continued TNFα induction. These results confirm that stimulation through TLR2 and NOD2 can regulate the ME inflammatory process in human middle ear cells. Our results further suggest that NOD2 is more involved in the recognition of NTHi PAMPs than NOD1 in HMEECs, mice and patients. Others (Woo et al., 2014) have also demonstrated that NOD2 recognition of NTHi produces human β-defensin 2 upregulation in HMEEC.

The most remarkable finding in our immunostimulation cell studies was that the double-stimulation with NTHi and either TNFα, Tri-DAP or MDP resulted in a decrease of TNFα gene expression. This hyporesponse is consistent with a negative feedback protective mechanism to strong immune reaction/stimulation. Such negative innate immune regulation has been noted by others. For example, peptidoglycan-activated NLRC4 interferes with the activation of NOD2, as a negative feedback regulator (Hruz and Eckmann, 2008).

Based on our results, inhibition of the inflammatory effects of TLR2, NOD2 and/or TNFα could be beneficial in reducing OM pathophysiology. Inhibitors of TLR2 (e.g (Grabowski et al., 2018)), NOD2 (Rickard et al., 2013) and TNF (Monaco et al., 2015) maybe useful therapeutics to explore. Obviously, given strong evidence that lack of the innate immune genes encoding these innate immune molecules leads to COM (Leichtle et al., 2009; Leichtle et al., 2010; Lee et al., 2019), any of these inhibitors would need to be used as adjuncts to antibiotic therapy, where they could be advantageous. They could ameliorate the inflammatory effects of PAMPs during active middle ear infection. Moreover, given that Eustachian tube dysfunction is often present in OM, they could also reduce inflammation due to dead bacteria and their components that persist in the middle ear due to poor drainage of the tympanic cavity.

In conclusion, this study elucidates interactions of key innate immune signaling pathways present in ME mucosal tissue and infiltrating cells, in response to individual and combined infections. Using a translational approach including cellular, animal model and clinical studies, we demonstrated that during COM, activation of TLR2-NOD2-TNF signaling axes (highlighted in **Figure 1**) controls the ME inflammatory response *via* a complex network of positive and negative regulators. This complexity provides opportunities for individual differences to modulate the mucosal response in OM, leading either to OM resistance or proneness. It also provides multiple opportunities for pharmacological intervention to reduce pro-inflammatory signaling or enhance negative regulation.

## Data Availability Statement

The data presented in the current study can be found at the Science Data Bank Online repository. https://www.scidb.cn/en/s/YR3M7n, DOI: 10.11922/sciencedb.01553.

## Ethics Statement

All protocols were approved by the ethics committee of the University of Lübeck. The patients/participants provided their written informed consent to participate in this study. The animal study was reviewed and approved by The Institutional Animal Care and Use Committee of the Veterans Affairs Medical Center in San Diego, CA.

## Author Contributions

AL and AK designed and supervised the project, designed experiments, conducted experiments and contributed to the writing of the manuscript. DL and MD performed qPCR, IHC and protein interaction. CD conducted animal experiments. AR and K-LB supported the execution of the experiments, editing the manuscript, and conducting the data analysis and interpretation. All authors contributed to the article and approved the submitted version.

## Funding

This work was supported by Grant E37-2010 (AL) University of Lübeck, the NIH Grant DC000129, and the VA Research Service-Grant 1BX001205 (AR), and NIH Grant DC014801 (AK).

## Conflict of Interest

AR is a co-founder of Otonomy Inc., serves as a member of the Scientific Advisory Board, and holds an equity position in the company. The UCSD Committee on Conflict of Interest has approved this relationship. Otonomy, Inc. played no part in the research reported here.

The remaining authors declare that the research was conducted in the absence of any commercial or financial relationships that could be construed as a potential conflict of interest.

## Publisher’s Note

All claims expressed in this article are solely those of the authors and do not necessarily represent those of their affiliated organizations, or those of the publisher, the editors and the reviewers. Any product that may be evaluated in this article, or claim that may be made by its manufacturer, is not guaranteed or endorsed by the publisher.

## Glossary

**Table:**

Bid	BH3 interacting-domain death agonist
Casp	Caspase
cCasp	cleaved caspase 3
COM	Chronic Otitis Media
CHX	Cycloheximid
EAS	External auditory skin
HMEEC	Human Middle Ear Epithelial Cell
IL8	Interleukin 8
KO mice	Knockout mice
MDP	Muramyl dipeptide
NLR	NOD-like receptor
NOD1/2	Nucleotide-binding oligomerization domain receptor1/2
ME	Middle ear
MyD88	Myeloid differentiation primary response 88
NTHi	Not typable Haemophilus influenza
OM	Otitis Media
PAMPS	Pathogen associated molecular patterns
PRR	Pattern Recognition Receptors
RIPK2	Receptor Interacting Serine/Threonine Kinase 2
SB203580	Pyridinyl imidazole and p38 MAP Kinase Inhibitor
TLR	Toll-like receptor
TNF and TNFα	Tumor necrosis factor (α)
Tri-DAP	L-Ala-γ-D-Glu-mDAP
URI	Upper respiratory infection
WT mice	Wild-type mice
