# Supplementary material for: Immunomodulation as a Protective Strategy in Chronic Otitis Media
Source: Front Cell Infect Microbiol. 2022 Mar 30;12:826192. doi: 10.3389/fcimb.2022.826192 (PMC9005906; doi:10.3389/fcimb.2022.826192)
Supplement: Supplementary file 1 [file Table_1.docx]

**Table S1:** Clinical and demographic information from patients utilized in this study.

(N=No and Y=yes)

COM:

| **Patient ID** | **Gender** | **Age (years)** | **COM** | **Healthy**  **MEM** | **EAS** | **Hearing Loss** | **Vertigo** | **Diagnosis/Surgery** |
| --- | --- | --- | --- | --- | --- | --- | --- | --- |
| 1 | F | 82 | Y | N | N | Y | N | COM with facial palsy |
| 4 | F | 67 | Y | N | N | Y | Y | COM with facial palsy |
| 13 | F | 53 | Y | N | N | Y | N | COM |
| 20 | F | 45 | Y | N | N | Y | N | COM |
| 21 | F | 62 | Y | N | N | Y | N | COM |
| 22 | F | 53 | Y | N | N | Y | N | COM |
| 28 | F | 38 | Y | N | N | Y | Y | COM with mastoiditis |
| 29 | F | 63 | Y | N | N | Y | N | COM |
| 30 | F | 23 | Y | N | N | Y | N | COM |
| 31 | F | 35 | Y | N | N | Y | Y | COM |
| 34 | F | 39 | Y | N | N | Y | N | COM |
| 37 | F | 52 | Y | N | N | Y | N | COM |
|  |  |  |  |  |  |  |  |  |
| 3 | M | 39 | Y | N | N | Y | N | COM |
| 6 | M | 22 | Y | N | N | Y | N | COM |
| 7 | M | 45 | Y | N | N | Y | N | COM |
| 8 | M | 69 | Y | N | N | Y | Y | COM |
| 11 | M | 64 | Y | N | N | Y | Y | COM with mastoiditis |
| 16 | M | 75 | Y | N | N | Y | N | COM |
| 26 | M | 52 | Y | N | N | Y | N | COM |
| 35 | M | 58 | Y | N | N | Y | Y | COM |

Healthy:

| 5 | F | 75 | N | Y | Y | Y | Y | acute Suditas for tympanoscopy |
| --- | --- | --- | --- | --- | --- | --- | --- | --- |
| 9 | F | 39 | N | Y | Y | Y | N | Cochlear implantation |
| 12 | F | 45 | N | Y | Y | Y | N | Vibrant Soundbridge implantation |
| 17 | F | 68 | N | Y | Y | Y | N | Cochlear implantation |
| 18 | F | 42 | N | Y | Y | Y | N | Cochlear implantation |
| 25 | F | 35 | N | Y | Y | Y | N | Cochlear implantation |
| 36 | F | 46 | N | Y | Y | N | N | Second-Look |
| 38 | F | 61 | N | Y | Y | Y | N | Vibrant Soundbridge implantation |
|  |  |  |  |  |  |  |  |  |
| 2 | M | 30 | N | Y | Y | Y | N | acute Suditas for tympanoscopy |
| 10 | M | 74 | N | Y | Y | Y | N | Cochlear implantation |
| 15 | M | 62 | N | Y | Y | Y | Y | acute Suditas for tympanoscopy |
| 19 | M | 59 | N | Y | Y | Y | N | Vibrant Soundbridge implantation |
| 23 | M | 49 | N | Y | Y | Y | N | acute Suditas for tympanoscopy |
| 24 | M | 56 | N | Y | Y | Y | N | Cochlear implantation |
| 27 | M | 80 | N | Y | Y | N | N | Cochlear implantation |
| 32 | M | 66 | N | Y | Y | N | N | Second-Look |
| 33 | M | 60 | N | Y | Y | Y | N | Vibrant Soundbridge implantation |
| 39 | M | 42 | N | Y | Y | N | N | Second-Look |
| 40 | M | 72 | N | Y | N | Y | N | Cochlear implantation |
| 41 | M | 67 | N | Y | Y | N | N | Second-Look |
| 14 | M | 57 | N | N | Y | N | N | Meatoplasty |
