# Supplementary material for: Immunomodulation as a Protective Strategy in Chronic Otitis Media
Source: Front Cell Infect Microbiol. 2022 Mar 30;12:826192. doi: 10.3389/fcimb.2022.826192 (PMC9005906; doi:10.3389/fcimb.2022.826192)
Supplement: Supplementary file 2 [file Table_2.docx]

**Table S2. Survey of several well-known innate immune receptors gene fold changes (median and range) after NTHi inoculation in the typical time course of OM. (P-values < 0.05 are in bold)**

| Time: | 0h | 3h | 6h | 24h | 2d | 3d | 5d | 7d |
| --- | --- | --- | --- | --- | --- | --- | --- | --- |
| **TLR2** | | | | | | | | |
| Fold Exp | 0.99 | **6.04** | **5.97** | **12.63** | 5.64 | 5.61 | 1.35 | 1.35 |
| Range | 0.88-1.12 | **5.45-6.69** | **5.32-6.7** | **11.28-14.13** | 4.48-7.14 | 4.81-6.53 | 1.27-1.44 | 1.29-1.41 |
| P-value | 0.96 | **0.04** | **0.04** | **0.03** | 0.08 | 0.05 | 0.13 | 0.09 |
| **TLR4** | | | | | | | | |
| Fold Exp | 0.99 | **1.24** | 1.34 | 3.08 | 1.91 | **2.71** | **1.88** | **2.17** |
| Range | 0.84-1.16 | **1.22-1.25** | 1.18-1.52 | 2.54-3.74 | 1.50-2.43 | **2.51-2.92** | **1.82-1.93** | **2.11-2.22** |
| P-value | 0.95 | **0.04** | 0.27 | 0.11 | 0.23 | **0.048** | **0.03** | **0.02** |
| TLR6 | | | | | | | | |
| Fold Exp | 0.99 | 2.17 | 3.16 | **8.20** | 4.02 | **4.28** | 1.72 | 1.29 |
| Range | 0.92-1.08 | 1.98-2.37 | 2.56-3.91 | **7.72-8.71** | 3.26-4.94 | **3.83-4.79** | 1.47-2.02 | 0.90-1.86 |
| P-value | 0.97 | 0.07 | 0.12 | **0.018** | 0.09 | **0.049** | 0.18 | 0.60 |
| TLR9 | | | | | | | | |
| Fold Exp | 0.813 | 0.80 | 0.65 | 1.02 | 2.53 | 0.78 | 1.93 | **1.475** |
| Range | 0.418-1.582 | 0.514-1.251 | 0.35-1.21 | 0.65-1.58 | 1.77-3.62 | 0.26-2.31 | 1.71-2.17 | **1.47-1.48** |
| P-value | 0.908 | 0.71 | 0.617 | 0.98 | 0.23 | 0.86 | 0.11 | **0.002** |
| **DAI** | | | | | | | | |
| Fold Exp | 0.974 | 4.06 | 3.17 | **12.35** | **41.43** | 28.43 | **9.08** | 1.66 |
| Range | 0.77-1.23 | 1.72-9.58 | 0.71-14.18 | **11.14-13.69** | **35.45-48.41** | 21.34-37.88 | **8.92-9.25** | 1.43-1.92 |
| P-value | 0.93 | 0.35 | 0.58 | **0.03** | **0.03** | 0.054 | **0.005** | 0.18 |
| POL-III | | | | | | | | |
| Fold Exp | 0.98 | 0.65 | 0.67 | **1.66** | 1.27 | 0.71 | 0.83 | 0.59 |
| Range | 0.82-1.18 | 0.6-0.72 | 0.62-0.71 | **1.63-1.70** | 1.18-1.38 | 0.55-0.92 | 0.71-0.96 | 0.55-0.64 |
| P-value | 0.94 | 0.14 | 0.10 | **0.03** | 0.2 | 0.41 | 0.43 | 0.09 |
| NOD1 | | | | | | | | |
| Fold Exp | 1.000 | 0.89 | 0.74 | **1.68** | 0.97 | 0.66 | 0.55 | 0.54 |
| Range | 0.997-1.003 | 0.81-0.92 | 0.7-0.78 | **1.66-1.7** | 0.89-1.05 | 0.6-0.73 | 0.50-0.60 | 0.53-0.54 |
| P-value | 0.999 | 0.26 | 0.12 | **0.01** | 0.78 | 0.15 | 0.09 | 0.08 |
| **NLRP3** | | | | | | | | |
| Fold Exp | 0.99 | **4.01** | 3.94 | **3.39** | 1.62 | 1.17 | **0.67** | 0.65 |
| Range | 0.88-1.12 | **3.74-4.30** | 2.82-5.51 | **3.27-3.51** | 0.91-2.91 | 0.72-1.91 | **0.65-0.69** | 0.44-0.96 |
| P-value | 0.96 | **0.03** | 0.15 | **0.02** | 0.56 | 0.79 | **0.05** | 0.46 |
|  | | | | | | | | |
